# Respecting Autonomy Over Time: Policy and Empirical Evidence on Re‐Consent in Longitudinal Biomedical Research

**DOI:** 10.1111/bioe.12165

**Published:** 2015-05-09

**Authors:** Susan E Wallace, Elli G Gourna, Graeme Laurie, Osama Shoush, Jessica Wright

**Keywords:** re‐consent, consent, autonomy, biobank, policy

## Abstract

Re‐consent in research, the asking for a new consent if there is a change in protocol or to confirm the expectations of participants in case of change, is an under‐explored issue. There is little clarity as to what changes should trigger re‐consent and what impact a re‐consent exercise has on participants and the research project. This article examines applicable policy statements and literature for the prevailing arguments for and against re‐consent in relation to longitudinal cohort studies, tissue banks and biobanks. Examples of re‐consent exercises are presented, triggers and non‐triggers for re‐consent discussed and the conflicting attitudes of commentators, participants and researchers highlighted. We acknowledge current practice and argue for a greater emphasis on ‘responsive autonomy,’ that goes beyond a one‐time consent and encourages greater communication between the parties involved. A balance is needed between respecting participants' wishes on how they want their data and samples used and enabling effective research to proceed.

## Introduction

The phrase, ‘*plus ça change, plus c'est la même chose*’ – loosely translated as ‘the more things change, the more they stay the same’^1^ – is particularly true when looking at the need to balance the autonomy of participants, as expressed through their consent to participate in biomedical research, with the fast pace of scientific enquiry. Consent, as it relates to longitudinal biomedical research has always been contentious. As these projects mature and the pressure increases at national and international levels to link resources to facilitate access and data sharing,^2^ there is a need to ensure that consent is valid and appropriate at every stage of the process and with respect to future possibilities.

Because longitudinal projects follow participants over time and provide access to resources for many years, change is inevitable. The objectives and science of the project, and the views of participants, may change. New uses of existing data may arise. All of these may cause projects to consider moving outside the scope of the original consent. Currently longitudinal projects generally use ‘broad’ consent for future unspecified research with appropriate governance mechanisms, a paradigm that is designed to be flexible and responsive. Consent should be an ongoing process that allows the research and the expectations of participants to move together. However, broad consent is not a complete answer, relying on governance mechanisms to decide on the acceptability of new propositions for study resources. At some tipping point, re‐consent needs to be an option in order to allow individuals to make their own decisions about participation.

## Methodology

The debates have so far mainly unfolded around the nature and potential applications of broad consent, and re‐consent has been underexplored. We conducted a systematic review of applicable policy statements and literature for the arguments for and against re‐consent from the perspective of longitudinal cohort studies, tissue banks and biobanks (hereafter ‘projects’). The following databases were searched: Web of Knowledge, Scopus, PubMed, the British Library Catalogue, Google Scholar, PMC, OpenGrey, official‐documents.gov.uk, PapersFirst, WorldCat and WorldCatDissertations. The search terms selected were: reconsent* OR re‐consent* AND research AND participant*. Searches using these terms were conducted between January 23 and February 5, 2013. Where possible, depending on the databases' search engines, the results returned were limited to display only results from 1990 onwards, as we judged re‐consent in this context to be a relatively recent activity. Results were reviewed for inclusion based on our research questions: What are seen as the benefits and disadvantages to re‐consent? What do people feel should and should not trigger re‐consent? What circumstances actually trigger a re‐consent exercise and what is the outcome? After reviewing our results we concluded that there was insufficient empirical data to present a systematic analysis. In addition, although there is considerable opinion on re‐consent, there were an insufficient number of comparable studies to allow us to draw supportable conclusions. Therefore a less constrained review is reported here, presenting the issues and identifying gaps in the research.

No agreed definition of re‐consent exists and there is considerable ambiguity in how it is described and used in practice. Re‐consent has been used to describe activities ranging from repeating the consent process, where refusal requires a participant to withdraw completely from the project, to asking for consent for a single new aspect of the project, refusal of which would allow continued participation. As we found insufficient evidence available to individually analyse each of these, for this article we define re‐consent broadly as the process of seeking participant consent to *change* or update their existing consent to allow their samples and data to be used in a different way from that which was originally agreed^3^ or to confirm that new uses fall within the expectations and understandings of participants. We have excluded re‐contact for administrative purposes that is agreed in the original consent, re‐consent in paediatric studies when a child comes of age, and in clinical trials when new information arises (e.g. drug safety) that may change the conduct of the trial or influence a participant's decision to participate. We present examples of re‐consent exercises, discuss triggers and non‐triggers for re‐consent and highlight the conflicting attitudes of commentators (i.e. academic and clinical professionals who have published the issues), participants and researchers. Finally, we argue for other alternatives, such as a greater emphasis on ‘responsive autonomy’, that go beyond one‐time consent and encourage greater communication between the parties involved *in both directions*.

## Results

### Current policy on re‐consent

Internationally, guidance from policy bodies is vague, focusing on re‐consent to ensure that a participant's consent remains informed if there are changes in the protocol or in the circumstances of the participant. For example, the Canadian Tri‐Council Policy Statement declares: ‘the researcher has an on‐going ethical and legal obligation to bring to participants' attention any changes to the research project that may affect them.’^4^ Similarly, the Council for International Organizations of Medical Sciences (CIOMS) notes, ‘[w]hen material changes occur in the conditions or the procedures of a study, and also periodically in long‐term studies, the investigator should once again seek informed consent from the subjects.’^5^ The US Office Human Research Protection suggests that consent may need to be ‘repeated’ or ‘supplemented’ if the protocol design or risks have changed or if a substantial period of time has elapsed since the original consent.^6^ Questions about length of time elapsed between consent and use of data is particularly pertinent for longitudinal research, but high‐level policy statements give little specific guidance on procedure. The Organization for Economic Co‐operation and Development (OECD) in its guidance for biobanks states that review practices, in accordance with applicable law and oversight mechanisms, should be in place ‘where human biological materials or data are to be used in a manner not anticipated in the original informed consent process.’^7^

There is little clarity regarding any legal requirements for re‐consent. At a European level, the Council of Europe (CoE) Additional Protocol to the Convention on Human Rights and Biomedicine, concerning Biomedical Research^8^ emphasizes that in case of new developments (i.e. additional scientific information becoming available regarding the research or changes in implications for participants), additional consent might be required. This protocol was, as of November 2014, signed by 22 member States, but only ratified by nine, with ratification necessary for integration into national legislation. The CoE previously published a recommendation (Rec(2006)4)^9^ suggesting that if the scope of the research is altered new consent should be sought. This is not legally binding and is currently under review, in the light of scientific developments and governance experiences.

Individual nation states offer little legal guidance and existing laws are not explicit. Laws in France and Italy^10^ state that re‐consent would be desirable if changes are made to the original research, but if this is judged to be impossible an authorization from the relevant authority should be sought. Similarly, in the US all research funded at any institution receiving federal funding has to abide by section 45 CFR 46.116(d) of the Code of Federal Regulations regarding ‘Protection of Human Subjects’^11^ which states that institutional review boards (IRBs) are responsible for deciding when re‐consent is required.

Initiatives, such as the Global Alliance for Genomics and Health, actively promote broad consent as a way to encourage sharing of results from research studies.^12^ Research funders have long supported sharing biomedical data but recognise that flexibility is needed as there are a ‘wide range of attitudes towards giving consent over time and for different research purposes’.^13^

### Current positions supporting or opposing re‐consent

Arguments around re‐consent are linked to discussions about informed consent more generally; specifically that consent at a single time‐point is inadequate for longitudinal projects because an individual cannot agree to future unknown uses of samples and data, and therefore consent is not informed throughout participation.^14^ Consent should not be seen as a ‘snapshot’ captured in a legal document, but as ‘the starting point of a relationship that will evolve over time’.^15^

Arguments in favour of re‐consent emphasize individual decision‐making. Seeking re‐consent when the reach of the original consent is in doubt gives participants greater autonomy and control over their samples and data^16^ and is thought to increase public trust in genomics research.^17^ Studies examining public viewpoints suggest that being asked for consent for each research use would enhance feelings of control, trust in the study, and would foster respect.^18^ Consent for specific uses could prevent misuse of resources, such as allowing research participants to refuse the use of their data in studies that may go against religious or moral beliefs.^19^ A strong relationship and frequent interaction between longitudinal research projects and participants is seen as beneficial for the project, fostering better relationships and increasing participant engagement.^20^ This allows participants to make autonomous decisions, including the right to withdraw, which is generally seen as absolute (subject to practical considerations when research resources have already been used up or transferred).

Arguments against re‐consent focus on disadvantages for the project, as well as for the participant. For the project, they centre on the impracticalities of the re‐consenting process, the high cost in terms of time and resources of finding and re‐consenting research participants^21^ and the reduction in the cohort size over time, leading to it being more difficult to conduct research.^22^ Re‐consent could introduce selection bias, as those who agree could be materially different from those who do not, thus potentially affecting the scientific validity of the data.^23^ If the consent becomes so specific, resulting in it only being able to be used for certain activities, this could limit the usefulness of the resource,^24^ if the original consent was determinedly broad in nature. One might also argue that re‐consent ‘cheapens’ broad consent, in that it suggests that existing governance mechanisms are insufficient.

‘Consent fatigue’ is often raised; re‐consent procedures may constitute a nuisance or loss of privacy, and may cause unnecessary distress for participants.^25^ Yet there is ambiguity when this position is explored. When cancer patients contributing to a tissue banking study were asked their preferences, ‘58% reported that re‐consent was a waste of time and money but 51.7% indicated they would feel respected and involved if asked to re‐consent.’^26^ As expressed by another study, ‘the request represented a tangible demonstration of the researchers' trustworthiness and regard.’^27^ This leads to questions of how much this reflects the impact of being asked for re‐consent, but also simply being given the opportunity to express one's preference. Thus, re‐consent is not a black‐and‐white process. Perhaps an examination of specific triggers can assist in determining when re‐consent is warranted, both for the participants and for projects.

### Triggers for re‐consent

Re‐consent is seen as not necessary in cases where there are only minor protocol changes that cause no perceived increased risk or concern to the participant^28^ or where the transaction and economic costs of seeking re‐consent would be disproportionate to the risk to participants or the project itself. The following changes may require re‐consent: major changes in protocol, researching a new unrelated condition, adding new genetic element, moving from phenotype‐driven research to whole genome sequencing (WGS), and ‘controversial’ research. These events could impact participants, both in terms of their privacy (re‐identification from genetic data) and their autonomy (new uses without their consent). Change without re‐consent could undermine the trust of participants and could cause researchers to suffer reputational damage. If projects, through the new use, discover genetic information that should be fed back to participants, there may be an impact on biological relatives and social families who have not consented directly to the research.

The longer a project continues the more likely there will be more significant changes in the protocol moving outside existing expectations and re‐consent may be needed. The UK Medical Research Council (MRC) Cognitive Function and Ageing Study re‐consented their participants because it was judged that the original consent did not adequately explain how samples and data were being used; this could be seen as increasing the risk to participants.^29^ Agreement was obtained from, or on behalf, of 197 of the 224 respondents (88%) it was possible to approach.^30^ Researchers from the UK International Cancer Genome Consortium (ICGC) Prostate Cancer project successfully re‐consented participants to allow samples and data to be used for ‘disease’ research, broadened from the original consent limited to ‘cancer’ research. In their report of January 2013, they state that in one recruitment site, 77 of 79 men approached agreed to the broader consent, with two men yet to answer. At a second site, 269 of 313 (86%) men agreed, and 44 (14%) declined.^31^ These results show that a strong majority of participants were happy to consent under these new terms. Such results can be interpreted as supporting Steinsbekk's argument that ‘there is no ethical difference between research on diabetes, cancer or cardiovascular disease’ if the research aims fall within the framework or ethos of the original consent.^32^ However, this position can only be supported if more is known about the original expectations of the participants, how closely the new use fits within that framework and whether the act of asking was the driver for agreement as opposed to a lack of concern about the new use.

When considering major protocol changes, the term ‘major’ needs to be examined in terms of how it differs from the original consent. The transfer of data and samples into a database that can be accessed internationally has raised questions, as this use might cause concerns regarding the adequate protection of privacy and security of data.^33^ One US project, the eMERGE (electronic MEdical Records and Genomics) Network, funded by the US National Institutes of Health (NIH), was required by their IRB to re‐consent participants in a longitudinal cohort study included in its multi‐institutional research collaboration.^34^ This re‐consent exercise was deemed as required because NIH funded studies must deposit their data into the federally‐administrated database of Genotypes and Phenotypes (dbGaP), an activity not covered in the original Adult Changes in Thought (ACT) consent. The re‐consent exercise could be deemed successful if the benchmark is a high rate of agreement: ‘of 1,340 cognitively intact study participants contacted for re‐consent, 1,159 (86%) agreed to participate in eMERGE and have their data deposited in dbGaP; 152 (11%) declined; and 29 (2%) were determined to be ineligible.’^35^

Adding a genetic component to an existing study is generally agreed by commentators to be substantial enough to warrant re‐consent, as it raises issues of re‐identifiability and the potential impact on biological relatives and social families who have not consented directly and may have no understanding of the implications of participation.^36^ A survey of genetics researchers and ethics review professionals found that the majority believed that re‐consent should also be sought when a new gene variant was to be studied or genetic measures added to a project.^37^ Researchers with HUNT 2 (the Nord‐Trøndelag Health Study) added a functional genomics component to its long‐running study, and contacted 61,426 participants via post and other media to ask for their consent.^38^ Only a small number of participants (1.9% or 1,185) withdrew their consent.^39^ Re‐consent was sought by the international MONICA (Monitoring trends and Determinants in Cardiovascular Disease) project, in 2001, to ask permission to use existing blood samples for ‘academic’ and ‘industrial’ genetic research. Of the 1409 participants, 93% agreed to academic genetic research and 91% agreed to industrial or commercial genetic research.^40^

A further move is from the broad category of ‘genetic research’ (e.g. the search for candidate genes) as presented to participants, to the more specific technology of whole genome sequencing (WGS). Professionals appear to see this as an increased risk to participants and are more inclined to see whole genome methods as adding extra complexity, given the potential for identifiability of the data and for incidental findings.^41^ All ICGC member studies are asked to obtain consent from their participants specifically for WGS; this is one of the core bioethical elements that need to be respected by members as a precondition of membership.^42^ Yet other commentators suggest that more advanced types of genetic research may be carried out without re‐consent if similar genetic research was described in the original consent, as the goals, risks and benefits may be similar and therefore within the expectations of participants.^43^

Similarly, DeCamp and Sugarman suggest that re‐consent should occur before research into any topics considered ‘controversial’, such as ‘violent behaviour’, as it cannot be assumed that every participant would agree to be involved.^44^ This obviously raises the question of what is controversial. Commercial use has been cited as such,^45^ yet the MRC Cognitive Function and Ageing Study discussed earlier allowed respondents or their families to opt‐out of the use of samples by commercial companies, but very few did,^46^ showing that commercial use was not, in this particular case, seen as controversial for all participants.

While one might assume that people would be risk‐adverse or have concerns, most participants in the studies we found do agree to the new uses, even for what is considered by many as a major or controversial change in the protocol. The question can be asked whether professionals and others are seeing hypothetical dangers that may not be troublesome for actual participants, or whether participants actually need to be protected from real dangers that they may not recognize or understand. Another question is whether the act of asking is the catalyst for acceptance, as individuals will decide differently what is controversial, risky or concerning to them. There is currently insufficient evidence to tease out the impact of each of these on the many other factors on which a decision might rest.

### Impact of re‐consent and alternatives

Even if most agree, requesting re‐consent can still have an impact on studies, particularly when examined longitudinally. Figures provided by the MONICA study show that it had lost 13% of its membership, primarily through non‐response or refusal by the end of its re‐consent exercise.^47^ Likewise, the ACT Study lost 14% of its participants.^48^ Repeated re‐consent will add to the losses suffered. Putting in place a procedure and then carrying out re‐consent can be a costly choice; even if only a very small number of people withdraw their consent, one needs to ask when the cost is justified.^49^

To counter these concerns, alternatives have been suggested. Anonymizing samples would negate the need for re‐consent and would enable data sharing, supporting altruistic participation, but does not answer privacy fears as anonymization is not a perfect protection and studies have shown that individuals can be re‐identified from genetic datasets in certain circumstances.^50^ Anonymization also negates the ability to withdraw from a study and should not be seen as a way to avoid ethical obligations to inform participants of any changes, participants could still experience distress in cases where their anonymized samples and data were used for reasons different from the ones to which they had consented.^51^

Using an opt‐out system has been suggested where re‐contact is possible and the risks of the new research were judged ‘minimal.’ Participants are notified by email, post or regular newsletter and be given the chance to opt‐out of specific research uses or withdraw completely.^52^ However, this paradigm may not be acceptable to participants. ACT participants overwhelmingly felt it was important that they be asked (90%); a significant minority (40%) did not agree with an opt‐out paradigm. Notification‐only was even less popular (67% disagreed), although the remaining third still found it acceptable according to their understanding of their original consent.^53^ This indicates the division of opinion and the need for further research. In one UK study, the general public suggested that their general practitioner (GP) could act as an intermediary between them and researchers, when re‐consent was sought.^54^ However, those surveyed also acknowledged the inherent difficulties, such as needing a stable relationship between researchers, participants and GPs, and the resource implications.^55^

The most common alternative being used is to make the decision in conjunction with an oversight body,^56^ such as an ethics committee.^57^ This aligns with a broad consent model with governance structures to protect the interests of participants. Ethics committees in countries such as the US have the ability to provide a waiver for re‐consent if certain criteria are met.^58^ Publishers are another group who act as decision‐makers. The editors of *PLOS Genetics* decided not to require 23andMe to re‐consent 3000 research participants due to the perceived practical problems involved in contacting those individuals. The editors did request that consent processes be amended in the future^59^ and the company agreed.

Technological options, such as patient‐centric initiatives, e.g. online interactive tools^60^ or dynamic consent models,^61^ have also been suggested. Enabling participants to review uses or agree to future research electronically could ameliorate concerns over the burden or impact of the re‐consent process. It can support broad consent, if the individual wishes to allow the study to make decisions on their behalf, but it also gives participants the opportunity to choose the access level with which they are most comfortable over time.^62^ Some authors believe that such a system should routinely be incorporated into new longitudinal studies.^63^

However, technological options will not always be appropriate, based on the difficulties of integrating them into existing projects and only some participants will be willing to engage on an individual level, which might affect representativeness. More importantly, such options take an individualistic stance on autonomy. This may not pay due attention to those who wish to participate from a position of solidarity, seeing participation as a public good. A balance is needed between those who wish to take an active part in decisions and those who are happy to let the project make decisions on their behalf. Taking a responsive autonomy approach would include discussions with all parties about the current state of the project, its future plans and what new issues it might face. It may help us to judge the relative impacts of a perception of increased risk, the pleasure in being asked, or annoyance in being bothered on re‐consent rates, leading to a more tailored re‐consent process. If communication is ongoing, and there is a better understanding of the expectations and needs of both participants and the project, re‐consent may not be required for every change in circumstance.

### Challenges for re‐consent

There are contradictory positions as to when changes require re‐consent. This is in part because those seeking answers are approaching the question from different angles with different stakeholders: the public who may never participate in a research study, those who are currently being approached for participation, healthy volunteers and participants. These attitudes cannot easily be reconciled; the public are answering hypothetical questions, while participants may be reacting from a position of knowledge. Also, previous work has shown that participants were generally more restrictive in their *hypothetical* preferences than in their *actual* response.^64^ Those in one study were ‘perplexed by the need for re‐consent, feeling that they had already provided such consent in the original wording.’^65^ This contrasts with studies where actual participants and the public, in hypothetical situations, preferred re‐consent for each new use.^66^ Actual participation and proximity appear to influence a person's perspective towards the necessity for re‐consent. Participants have been shown to be happy to allow the project to make the decision if there is belief and interest in the subject being researched and trust in the project and its leaders.^67^ However, not all participants may be as trusting. Studies examining this wider issue of trust between researchers and participants have found similar diverging views.^68^

Professionals appear to be more cautious regarding what should require re‐consent as compared to the actual evidence from re‐consent exercises. New genetic methodologies are judged as needing re‐consent by professionals, yet when approached, a majority of participants say yes, leading us to ask if participants see potential risks in a different way or were reacting to the opportunity to state a preference. In general, studies have shown that people are quite happy with broad consent^69^ leading some participants to wonder why researchers ‘are just not getting on with the research.’^70^ Respecting the autonomy and wishes of participants is difficult when there seems to be no clear consensus across groups.

This calls into question what, in reality, we are trying to protect or promote through re‐consent. ‘Autonomy’, or self‐determination in the well‐worn neo‐liberal sense, is the glib answer. But what *type* of autonomy is being perpetuated in an approach to re‐contact or re‐consent a research participant in the long‐term projects interest us here? We note, for example, that many of the justifications for re‐approaching participants are driven by a lack of knowledge or understanding about participants, or better *between* participants and researchers. In many of the re‐consent scenarios discussed or envisioned in the literature, participants are essentially passive; they are cast as disempowered players on the research stage who must be empowered with new knowledge and new options. All of the responsibility for determining whether re‐consent should occur is placed on the researchers and their governance mechanisms. The communication is unidirectional, and not always welcome, as we have seen above. This means that researchers and those who govern them often have to second‐guess participants' perspectives and how they themselves might view their own autonomy. A common framework for such deliberations is to ask: what would a reasonable or responsible participant need or want to know? But it is the participant alone who is excluded from such musings. It removes the self‐ from self‐determination.

Equally, the retort cannot be: ‘just ask’, because this would require approaches in the case of all new uses, and this is neither necessary nor proportionate, nor ironically, would it promote autonomy in all cases (since some people would resent the approach). We suggest that what is required in these cases is a genuine sharing of information and power in the relationship *throughout its course*, and from the participant's perspective there should be more promotion of the concept of ‘responsive autonomy’. For us, this embraces the idea that with autonomy comes responsibility. In this context, it would be a responsibility for researchers to engage actively with the participant community about options and proposals, e.g. through a study website or newsletter. For participants it would be a responsibility to engage in this process, providing feedback and comment on said options and proposals. Responsive autonomy would promote a dialogue between researchers about the aims of the study and the future of the science, with participants who can discuss their understandings and expectations. Such discussions could help make clearer the bases on which decisions on re‐consent are made. Open and on‐going two‐way communication, as part of a responsive autonomy approach, on issues that might be ground‐breaking and therefore unknown and potentially ‘scary’ might help to disabuse concerns and pre‐empt the need for re‐consent.

## Conclusion

The shifting nature of science highlights the ever‐present struggle to balance the pursuit of research outcomes with participants' rights to make autonomous decisions about research uses of their data and samples. It is a challenge to balance the desire for control with the desire to see research progress; it is a greater challenge when the public, participants, patients and experts hold different opinions and no consensus can be reached.

Our study is limited in that there are insufficient studies with each of these groups in order to clearly define when and for what reasons re‐consent should be undertaken. We advocate the continued collection of evidence from re‐consent exercises and recommend the development of best practice guidelines. Also, most of the projects discussed were conducted in the UK and the US and we would welcome more evidence from other groups internationally, as these will reflect different cultural perspectives towards the issues. At this time we risk making judgements that cannot be generalized across countries.

In summary, some points are clear. Re‐consent policies need to be considered when a project is designed and part of the original consent. The original consent must be seen as setting a baseline of the expectations of both the participant and the project, and a benchmark against which future re‐consent decisions are based. Consent is an ongoing responsive process of communication, showing respect for autonomy and is not a series of yes or no answers. Re‐consent may be minimized if this is borne in mind throughout the project. Consent is not, on the other hand, the *same* as respect for autonomy. Participants should be able to opt‐out of projects if they wish; equally they should be able to consent and leave the future decisions to others. There is a clear recognition by the public and participants that re‐consent can be onerous, but they still see the value in being asked. Whether this can ever be reconciled is unknown but needs to be explored.

When change occurs, re‐consent can show respect to participants, as well as, ‘to the institution of consent’.^71^ But attention also needs to be paid to the desire shown by people to contribute to research and to the scientific studies seeking to improve people's lives and health. A ‘happy medium’ is needed.

## Footnotes

1

B.A. Karr . Epigram. Les Guêpes. 1849.

2

European Science Foundation . 2007 Shared Responsibilities in Sharing Research Data: Policies and Partnerships. Available at: http://www.esf.org/fileadmin/Public_documents/Publications/SharingData_01.pdf [Cited 2014 May 10].

3

E.J. Ludman , S.M. Fullerton , L. Spangler , S.B. Trinidad , M.M. Fujii , G.P. Jarvik , et al. Glad you Asked: Participants' Opinions of Re‐consent for Dbgap Data Submission. J Empir Res Hum Res Ethics 2010; 5: 9–16, p.12.2083141710.1525/jer.2010.5.3.9PMC3071850

4

Canadian Institutes of Health Research, Natural Sciences and Engineering Research Council of Canada, and Social Sciences and Humanities Research Council of Canada . Tri‐Council Policy Statement – Ethical Conduct for Research Involving Humans. 2010, p.33–34.

5

Council for International Organizations of Medical Science . International ethical guidelines for biomedical research involving human subjects. Available at http://www.cioms.ch/publications/layout_guide2002.pdf [Accessed 20 November 2014].14983848

6

Office for Human Research Protections . Informed Consent. Available at: http://www.hhs.gov/ohrp/policy/consentckls.html [Accessed 10 May 2014].

7

Organization for Economic Cooperation Development . OECD Guidelines on Human Biobanks and Genetic Research Databases. Eur J Health Law 2010; 17: 191–204, p.198.20443450

8

Council of Europe . Additional Protocol to the Convention on Human Rights and Biomedicine, concerning Biomedical Research. 2005 Available at: http://conventions.coe.int/Treaty/en/Treaties/Html/195.htm [Accessed 24 November 2014].

9

Council of Europe . Recommendation 4 on Research on Biological Materials of Human Origin. 2006.

10

Republique Francaise . Code De La Santé Publique – Loi du Aout 2004. 2004; Gazzetta Ufficiale n. 52 del 2‐3‐2012. Available at: http://www.altalex.com/index.php?idnot=17432 [Cited 2014 January 29].

11

Department of Health and Human Services , Federal Policy for the Protection of Human Subjects (‘Common Rule’), 45 CFR 46.116(d). 2009 Available at: http://www.hhs.gov/ohrp/humansubjects/commonrule/ [Cited 2014 May 5].

12

Global Alliance for Genomics and Health . 2014 Available at: http://www.globalalliancepr.org/ [Cited 2014 June19].

13

Wellcome Trust . Nuffield Council on Bioethics: The Linking and Use of Biological and Health Data – Response by the Wellcome Trust. 2014, p.11.

14

M.F.A. Otlowski . Tackling Legal Challenges Posed By Population Biobanks: Reconceptualising Consent Requirements. Med Law Rev 2012; 20: 191–126.2229073010.1093/medlaw/fwr035

15

G. Laurie & E. Postan . Rhetoric or Reality: What is the Legal Status of the Consent Form in Health‐Related Research? Med Law Rev 2013; 21: 371–414, p.371–372.2305557210.1093/medlaw/fws031PMC3746797

16

B.E. Ellerin , R.J. Schneider , A. Stern , P.G. Toniolo & S.C. Formenti . Ethical, Legal, and Social Issues Related to Genomics and Cancer Research: the Impending Crisis. J Am Coll Radiol 2005; 2: 919–926.1741196610.1016/j.jacr.2005.03.016

17

Otlowski *op. cit.* note 14; Ellerin et al., *op. cit* note 16; Phoebe . Harmonising Biobank Research: Maximising Value – Maximising Use, March 2009 Conference Summary. 2009; M.A. Igbe & C.A. Adebamowo . Qualitative Study of Knowledge and Attitudes to Biobanking Among Lay Persons in Nigeria. BMC Medical Ethics 2012; 13;2307232110.1186/1472-6939-13-27PMC3507723 A.L. McGuire , T. Caulfield & M.K. Cho , Science and Society – Research Ethics and the Challenge of Whole‐Genome Sequencing. Nature reviews Genetics 2008; 9: 152–156.10.1038/nrg2302PMC222544318087293

18

J. Murphy , J. Scott , D. Kaufman , G. Geller , L. LeRoy & K. Hudson . Public Perspectives on Informed Consent for Biobanking. Am J Public Health 2009; 99: 2128–2134.1983398810.2105/AJPH.2008.157099PMC2775766

19
Ibid.

20

S.B. Trinidad , S.M. Fullerton , E.J. Ludman , G.P. Jarvik , E.B. Larson & W. Burke . Research ethics. Research Practice and Participant Preferences: the Growing Gulf. Science 2011; 331: 287–288;2125233310.1126/science.1199000PMC3044500 V. Árnason . Scientific citizenship, benefit, and protection in population‐based research. Ethics Res Biob 2009: 131–141.

21

Global Alliance *op. cit.* note 12; Otlowski *op. cit* note 14; Ellerin et al. *op. cit* note 16; T. Caulfield , A.L. McGuire , M. Cho , et al. Research ethics recommendations for Whole‐Genome Research: Consensus Statement. PLoS Biology 2008; 6: 73;10.1371/journal.pbio.0060073PMC227032918366258 C.A. McCarty , A. Garber , J.C. Reeser & N.C. Fost . Personalized Med Res P, Ethics Security Advisory B. Study Newsletters, Community and Ethics Advisory Boards, and Focus Group Discussions Provide Ongoing Feedback for a Large Biobank. Am J Med Genet A 2011; 155A: 737–741.2157288910.1002/ajmg.a.33896PMC3092645

22

McCarty et al. *op. cit.* note 21; O.F. Bathe & A.L. McGuire . The Ethical Use of Existing Samples for Genome Research. Genet Med 2009; 11: 712–715;1974575010.1097/GIM.0b013e3181b2e168 K.L. Edwards , A.A. Lemke , S.B. Trinidad , et al. Attitudes Toward Genetic Research Review: Results from a Survey of Human Genetics Researchers. Public health genomics 2011; 14: 337–345.2148721110.1159/000324931PMC3221257

23

M.G. Hansson . Building on Relationships of Trust in Biobank Research. J Med Ethics 2005; 31: 415–418;1599436310.1136/jme.2004.009456PMC1734187 International Cancer Genome Consortium . Goals, Structure, Policies and Guidelines 2008. Available at: https://icgc.org/icgc/goals‐structure‐policies‐guidelines [Accessed 15 February 2014].

24

K.S. Steinsbekk & B. Solberg . Biobanks‐When is Re‐consent Necessary? Public Health Ethics 2011; 4: 236–250.

25

Árnason *op. cit.* note 20; Bathe & McGuire *op. cit* note 22; M. DeCamp & J. Sugarman . Ethics in Behavioral Genetics Research. Account Res 2004; 11: 27–47.1534104510.1080/725289013

26

Z. Master , J.O. Claudio , C. Rachul , J.C. Wang , M.D. Minden & T. Caulfield . Cancer Patient Perceptions on the Ethical and Legal Issues Related to Biobanking. BMC Med Genomics 2013; 6: 8, p.1.2349770110.1186/1755-8794-6-8PMC3599691

27

Trinidad et al., *op. cit* note 20, p.3.

28

McCarty et al., *op. cit.* note 21; Bathe & McGuire , *op. cit.* note 22.

29

T.L. Beauchamp . Informed Consent: its History, Meaning, and Present Challenges. Camb Q Healthc Ethics 2011; 20: 515–523.2184338210.1017/S0963180111000259

30

L. Barnes , F.E. Matthews , B. Barber , L. Davies , D. Lloyd , C. Brayne , et al. Brain Donation For Research: Consent and Re‐Consent Post *Alder Hey*
. Bull Med Ethics 2005; 211: 17–21.17139813

31

C. Cooper & R. Eeles . ICGC First Annual Report: Prostate Cancer 2013.

32

Steinsbekk & Solberg , *op. cit.* note 24, p.238.

33

Ludman et al., *op. cit.* note 3; E. Vermeulen , M.K. Schmidt , N.K. Aaronson , M. Kuenen & F.E. van Leeuwen . Obtaining ‘Fresh’ Consent for Genetic Research with Biological Samples Archived 10 Years Ago. Eur J Cancer 2009; 45: 1168–1174.1923248610.1016/j.ejca.2008.11.022

34

Ludman et al., *op. cit.* note 3, p. 4.

35
Ibid: 4.

36

J. Holmen , K. Midthjell , Ø Krüger , A. Tverdal , T. Claudi , A. Bjorndal , et al. The Nord‐Trøndelag Health Study 1995–97 (HUNT 2): Objectives, Contents, Methods and Participation. Norsk Epid 2003; 13: 19–32.

37

Edwards et al., *op. cit.* note 22.

38

Holmen et al., *op. cit.* note 36.

39
Ibid.

40

B. Stegmayr & K. Asplund . Informed Consent for Genetic Research on Blood Stored for More Than a Decade: a Population Based Study. BMJ 2002; 325: 634–635.1224217510.1136/bmj.325.7365.634PMC126305

41

J. Kaye , P. Boddington , J. de Vries , N. Hawkins & K. Melham . Ethical implications of the use of whole genome methods in medical research. Eur J Hum Genet 2010; 18: 398–403.1988829310.1038/ejhg.2009.191PMC2879322

42

International Cancer Genome Consortium , *op. cit* note 23.

43

H.K. Tabor , T. Brazg , J. Crouch , E.E. Namey , S.M. Fullerton , L.M. Beskow , et al. Parent Perspectives on Pediatric Genetic Research and Implications For Genotype‐Driven Research Recruitment. J Empir Res Hum Res Ethics 2011; 6: 41–52.2222805910.1525/jer.2011.6.4.41PMC3426316

44

DeCamp & Sugarman , *op. cit.* note 25, p.28.

45

Steinsbekk & Solberg , *op. cit.* note 24.

46

Barnes & Matthews , *op. cit.* note 30.

47

Stegmayr & Asplund , *op. cit* note 40.

48

Ludman et al., *op. cit* note 3.

49

L. Johnsson , M.G. Hansson , S. Eriksson & G. Helgesson . Patients' Refusal to Consent to Storage and Use of Samples in Swedish Biobanks: Cross Sectional Study. BMJ 2008; 337: 345.10.1136/bmj.a345PMC265692518617496

50

E. Schadt , S. Woo & K. Hao . Bayesian Method to Predict Individual SNP Genotypes from Gene Expression Data. Nat Genet 2012; 44: 603–608.2248462610.1038/ng.2248

51

D. Beyleveld & D. Townend . When is Personal Data Rendered Anonymous? Interpreting Recital 26 of Directive 95/46/EC. Med Law Int 2004; 6: 73–86.1662296410.1177/096853320400600201

52

D. Wendler & E. Emanuel . The Debate over Research on Stored Biological Samples: What do Sources Think? Arch Intern Med 2002; 162: 1457–1462;1209088110.1001/archinte.162.13.1457 G. Haddow , A. Bruce , S. Sathanandam , J.C. Wyatt . ‘Nothing is Really Safe’: a Focus Group Study on the Processes of Anonymizing and Sharing of Health Data for Research Purposes. J Eval Clin Pract 2010; 17: 1140–1146.2062999710.1111/j.1365-2753.2010.01488.x

53

Ludman et al. *op. cit* note 3.

54

Haddow et al. *op. cit.* note 52.

55

V. Armstrong , Wellcome Trust . Public Perspectives on the Governance to Biomedical Research: A Qualitative Study in a Deliberative Context. Wellcome Trust; 2006.

56

T. Caulfield & B.M. Knoppers . Consent, Privacy & Research Biobanks – Policy Brief No. 1. Genome Canada; 2010.

57

Steinsbekk & Solberg , *op. cit.* note 24; DeCamp & Sugarman , *op. cit* note 25.

58

Office for Human Research Protections *op. cit.* note 11.

59

G. Gibson & G.P. Copenhaver . Consent and Internet‐Enabled Human Genomics. PLoS Genet. 2010; 6.10.1371/journal.pgen.1000965PMC289170120585615

60

N. Anderson & K. Edwards . Building a Chain of Trust: Using Policy and Practice to Enhance Trustworthy Clinical Data Discovery and Sharing. 2010.

61

J. Kaye , E.A. Whitley , D. Lund , M. Morrison , H. Teare & K. Melham . Dynamic Consent: a Patient Interface for Twenty‐First Century Research Networks. Eur J Hum Genet 2014.10.1038/ejhg.2014.71PMC413065824801761

62

Anderson & Edwards , *op. cit.* note 60.

63

S.M. Fullerton , N.R. Anderson , G. Guzauskas , D. Freeman , K. Fryer‐Edwards . Meeting the Governance Challenges of Next‐Generation Biorepository Research. Science Tran Med 2010; 2: 15cm13;10.1126/scitranslmed.3000361PMC303821220371468 N. Anderson , C. Bragg , A. Hartzler & K. Edwards . Participant‐Centric Initiatives: Tools to Facilitate Engagement in Research. Appl Transl Genomics 2012; 1: 25–29;10.1016/j.atg.2012.07.001PMC399872424772384 C.M. Simon , J.K. Williams , L. Shinkunas , D. Brandt , S. Daack‐Hirsch & M. Driessnack . Informed Consent and Genomic Incidental Findings: IRB Chair Perspectives. J Empir Res Hum Res Ethics 2011; 6: 53–67.2222806010.1525/jer.2011.6.4.53PMC3616513

64

J.M. Oliver , M.J. Slashinski , T. Wang , P.A. Kelly , S.G. Hilsenbeck & A.L. McGuire . Balancing the Risks and Benefits of Genomic Data Sharing: Genome Research Participants' Perspectives. Public Health Genomics 2012; 15: 106–114.2221378310.1159/000334718PMC3318928

65

Barnes et al., *op. cit.* note 30, p. 19.

66

Murphy et al., *op. cit.* note 18; A. Tupasela , A. Sihvo , K. Snell , P.A. Jallinoja , A.R. Aro , et al. Attitudes Towards Biomedical Use of Tissue Sample Collections, Consent, and Biobanks Among Finns. Scand J Public Health 2010; 38: 46–52;1990677210.1177/1403494809353824 Master et al., *op. cit* note 26; C.M. Halverson & L.F. Ross . Incidental Findings of Therapeutic Misconception in Biobank‐Based Research. Genet Med 2012; 14(6): 611–615.2226176010.1038/gim.2011.50PMC4251740

67

Master et al., *op. cit.* note 26.

68

M. Dixon‐Woods , R.E. Ashcroft , C.J. Jackson , et al. Beyond ‘Misunderstanding’: Written Information and Decisions about Taking Part in a Genetic Epidemiology Study. Soc Sci Med 2007; 65: 2212–2222.1790471610.1016/j.socscimed.2007.08.010

69

Armstrong & Wellcome Trust , *op. cit.* note 55; Simon et al. *op. cit.* note 63.

70

Vermeulen et al., *op. cit* note 33.

71

Steinsbekk et al. *op. cit.* note 24, p.238.

